# Complementing the case for standardized travel health competencies: a UK perspective

**DOI:** 10.1093/jtm/taaf094

**Published:** 2025-09-09

**Authors:** Oliver Koch, Jane Chiodini, Amy Gannon, Mark Stephen Bailey, Samuel H Allen, Anne Mclean, David Andrew Ross

**Affiliations:** Regional Infectious Diseases Unit, NHS Lothian, Edinburgh, UK; Faculty of Travel Medicine, Royal College of Physicians and Surgeons of Glasgow, Glasgow, UK; Faculty of Travel Medicine, Royal College of Physicians and Surgeons of Glasgow, Glasgow, UK; Faculty of Travel Medicine, Royal College of Physicians and Surgeons of Glasgow, Glasgow, UK; Faculty of Travel Medicine, Royal College of Physicians and Surgeons of Glasgow, Glasgow, UK; Faculty of Travel Medicine, Royal College of Physicians and Surgeons of Glasgow, Glasgow, UK; Faculty of Travel Medicine, Royal College of Physicians and Surgeons of Glasgow, Glasgow, UK

To the Editor,

We read with great interest the article by Hess et al.^1^ published in 2024, and issued in December 2024, which outlined the current landscape of multidisciplinary travel health education and advocating for the development of standardized competencies. The authors rightly highlight the variability in training across professions and jurisdictions. However, we wish to draw attention to several UK-based resources and programmes that complement and reinforce the authors’ aims.

The Faculty of Travel Medicine (FTM) of the Royal College of Physicians and Surgeons of Glasgow published its Good Practice Guidance for Providing a Travel Health Service (2020).^2^ This peer-reviewed document outlines minimum standards of care, principles of governance, and includes a validated competency assessment tool. While developed in a UK context, the guidance is intended to be applicable internationally and is used by practitioners across a wide range of countries. The FTM’s international membership reflects the global relevance of its work and the shared commitment to raising standards in travel health practice worldwide.

Similarly, the Royal College of Nursing’s Travel Health Nursing: Career and Competence Development (2023, 4th ed.)^3^ offers a structured, career-spanning competency framework for nurses. Widely used within the UK, this document has also influenced similar guidance in countries such as Australia, the Netherlands, and Japan, and is referenced by UK regulatory bodies including the Care Quality Commission.

In addition, we note two established UK-based educational programmes that directly address many of the educational gaps identified in the article:

- The RCPSG Postgraduate Diploma in Travel Medicine, delivered in collaboration with Glasgow Caledonian University, provides an academically rigorous pathway to Faculty membership.
- The Professional Diploma in Travel Health, developed by the Liverpool School of Tropical Medicine in partnership with the National Travel Health Network and Centre (NaTHNaC), offers structured, multidisciplinary online training aligned with defined competencies.

These programmes exemplify best practice in travel health education and have been designed to support consistent, high-quality care across professional groups from a variety of national and international backgrounds.

We would also like to acknowledge the foundational publication ‘Recommendations for the practice of travel medicine’ by Chiodini et al.,^4^ which articulates a comprehensive knowledge base and continues to be highly relevant in shaping travel health practice and education.

Rather than offering a critique, we hope this letter highlights opportunities for greater alignment and collaboration. Recognizing and building upon existing frameworks such as these can help accelerate progress towards globally consistent, multidisciplinary travel health competencies.
